# Malocclusion and orthodontic treatment needs among individuals with down syndrome in Egypt: a multicentre cross-sectional study

**DOI:** 10.1186/s12903-026-09995-9

**Published:** 2026-10-19

**Authors:** Hani AlSwafeeri, Nancy Bedwani, Mariam Badran, Nada Shehata, Hala Ahmed, Menna Allah Mohamed

**Affiliations:** 1 Department of Orthodontics, Faculty of Dentistry, Pharos University, Alexandria, Egypt; 2 Department of Dental Public Health, Faculty of Dentistry, Pharos University, Alexandria, Egypt; 3 Faculty of Dentistry, Pharos University, Alexandria, Egypt

**Keywords:** Down syndrome, Malocclusion, Orthodontic treatment need, Special needs dentistry

## Abstract

**Background:**

Individuals with Down syndrome commonly present with a variety of craniofacial and dental anomalies that increase the prevalence of malocclusion and the need for orthodontic treatment. Despite this, data regarding orthodontic needs in this population in the middle east, particularly in Egypt, remain limited.

**Aim of the study:**

This study aimed to assess the patterns of malocclusion and orthodontic treatment needs among individuals with Down syndrome.

**Materials and methods:**

A total of 117 individuals with Down syndrome aged 12–25 years, who met the study’s eligibility criteria, were recruited from specialized institutions in Egypt in a multi-centred cross-sectional study. Clinical examination included extraoral and intraoral assessment, occlusal analysis using Angle’s classification, and assessment of the need for orthodontic treatment using Dental Health Component of Index of Orthodontic Treatment Need (IOTN). Additional data on oral hygiene, oral habits, and craniofacial features were recorded. Descriptive statistics were computed for all study variables. Qualitative (categorical) variables were presented as frequencies (n) and percentages (%).

**Results:**

Class I molar relationship was the most common (41.0%), followed by Class II (29.9%) and Class III (29.1%). Anterior open bite (23.1%) and posterior crossbite (21.4%) were also frequently observed. Regarding orthodontic treatment need, 71.7% of participants demonstrated borderline to very great need (IOTN Grades 3–5), with 49.6% exhibiting great or very great need (Grades 4–5). Fair or poor oral hygiene was identified in 81.2% of participants. Mouth breathing was the most common oral habit (32.5%).

**Conclusion:**

Patients with Down syndrome exhibit a high prevalence of malocclusion and a significant need for orthodontic treatment. Early orthodontic assessment, preventive strategies, and multidisciplinary management are essential to improve oral health outcomes in this population.

**Clinical trial number:**

Not applicable.

**Supplementary Information:**

The online version contains supplementary material available at https://doi.org/10.1186/s12903-026-09995-9.

## Introduction

Individuals who require additional care and attention in healthcare settings are commonly referred to as individuals with special healthcare needs [1, 2]. This group includes both children and adults who present with medical, physical, or intellectual conditions that may limit their ability to receive routine dental care. Such limitations often involve difficulties in communication, reduced cooperation, or physical constraints that complicate standard clinical procedures [3, 4].

Among these conditions, Down syndrome (DS), also known as trisomy 21, is one of the most prevalent genetic disorders. It results from the presence of a complete or partial extra copy of chromosome 21 [5]. This condition is associated with characteristic craniofacial features, delayed physical growth, varying degrees of intellectual disability, and an increased susceptibility to several systemic and oral health conditions. Early intervention and appropriate multidisciplinary care can significantly improve the quality of life and functional outcomes of affected individuals.

From a dental perspective, individuals with Down syndrome commonly exhibit a range of oral and craniofacial anomalies. These include malocclusion, delayed eruption of teeth, congenitally missing teeth, discrepancies in tooth size, impacted or transposed teeth, tongue thrusting habits, and a protrusive tongue posture. Additionally, skeletal discrepancies such as maxillary hypoplasia in both anteroposterior and transverse dimensions are frequently observed [6, 7]. These factors collectively contribute to a higher prevalence of orthodontic problems within this population.

A recent meta-analysis by Vicente et al. highlighted that reduced maxillary dimensions in individuals with Down syndrome restrict upper arch development, directly predisposing patients to a severe skeletal Class III malocclusion and posterior crossbite. This deficient maxillary growth, compounded by a relatively large tongue and habit patterns like tongue thrusting, frequently causes an anterior open bite where the opposing front teeth fail to meet. Furthermore, underlying T-cell deficiencies and impaired neutrophil function drastically reduce the body’s defense mechanisms against oral bacteria, leading to an exceptionally high prevalence of early-onset, aggressive periodontal disease even in the absence of heavy plaque accumulation [8].

Individuals with Down syndrome exhibit a high prevalence of severe skeletal malocclusions and an urgent need for orthodontic intervention compared to non-syndromic populations [9, 10]. Angle Class III malocclusion is the most dominant sagittal trait, affecting approximately 59% of individuals across both pediatric and cross-sectional global cohorts [11]. Affected individuals also present with distinct morphological differences, including significantly elevated rates of missing teeth (56.2%), incisal spacing (73.0%), and anterior maxillary irregularity (95.5%) compared to healthy controls [9]. Consequently, objective clinical indices reveal that between 81.9% and 85.3% of these patients present with an absolute, mandatory need for orthodontic care [10, 11].

However, significant barriers to successful treatment persist, including a widespread 30.4% lack of specialist referrals and a high 45% premature treatment termination rate due to patient compliance issues and severe hypodontia [11, 12].

Previous studies have highlighted the significant need for orthodontic intervention among individuals with Down syndrome. For instance, a cross-sectional study conducted by Alkawari [13] assessed orthodontic treatment needs using the Index of Orthodontic Treatment Need (IOTN) and reported that 81.9% of participants required treatment. Among these, Class III malocclusion was the most prevalent (59.1%), followed by Class I occlusion (36.4%). No statistically significant differences were observed between genders in relation to treatment need.

Despite the high prevalence of dental anomalies, routine dental examination and management of individuals with Down syndrome can be challenging for both caregivers and dental professionals due to behavioral and medical considerations. Additionally, there is a lack of research comprehensively linking malocclusion patterns, treatment needs, oral health, and craniofacial features across varied Down syndrome cohorts. Furthermore, there is a distinct lack of comprehensive research assessing malocclusion patterns and orthodontic intervention priorities specifically tailored to Down syndrome populations in Egypt and the broader Middle East. Therefore, a comprehensive understanding of their specific needs is essential to provide appropriate and effective dental care. Consequently, there is a compelling need to investigate these specific regional manifestations, serving as a vital clinical foundation to optimize orthodontic care delivery for this underserved population.

### Aim of the study

The aim of this cross-sectional observational study was to assess the prevalence, patterns, and severity of malocclusion, oral health status, and craniofacial features and orthodontic treatment needs among individuals with Down syndrome in Egypt.

This aim was achieved through the following approaches:

1. To clinically characterize malocclusion in patients with Down syndrome using Angle’s classification and the Dental Health Component (DHC) of the Index of Orthodontic Treatment Need (IOTN).
2. To clinically assess occlusal relationships and dental arch morphology utilizing a non-invasive approach for such special needs group. This was accomplished through the use of digital intraoral scanning technology.
3. To systematically evaluate oral health status, including oral hygiene, using Oral Hygiene Index-Simplified (OHI-S) as well as the presence of deleterious oral habits associated with malocclusion development and progression among the same target group.
4. To characterize associated craniofacial features among patients with Down syndrome, including facial type, profile analysis, facial profile, facial divergence, lip competence, or nasolabial angle through standardized extra-oral examination.

## Materials and methods

### Study design

This investigation was designed as an observational, cross-sectional study conducted over a three-month period from January 2026 to April 2026. The study aimed to evaluate the prevalence, patterns, and severity of malocclusion and orthodontic treatment needs among individuals with Down syndrome attending specialized care institutions.

The study was carried out across multiple specialized institutions in Alexandria and Beheira governorates, Egypt, including Gam’iyat Al-Hayah School, El Tarbeya El Fekreya School and Fairhaven School. Research was conducted at such centers based on accessibility and availability of eligible participants.

To ensure methodological rigor, reliability, and consistency of clinical assessments, all examinations were conducted by four well trained calibrated examiners, orthodontist and community dentist with experience in special needs populations. Comprehensive calibration sessions were conducted on 20 participants prior to the initiation of data collection to standardize diagnostic criteria, particularly concerning the application of the Index of Orthodontic Treatment Need (IOTN) and Angle’s classification system. Calibration participants were included in the final sample. Inter-examiner reliability was assessed using kappa statistics, with values exceeding 0.85 indicating excellent agreement.

### Target population/sample size

Based on a previous study by Alkawari H [13] showing expected prevalence of 81.9%, and using margin of error of 7% and confidence level of 95%, the estimated minimum required sample size was 117 participants. The sample size was calculated using Epi-info software (Epi-info software for Windows, Version 7.2.6, StatCalc Module, Centers for Disease Control and Prevention, Atlanta, Georgia).

### Study participants

An initial sample of 200 individual with Down syndrome was assessed for eligibility. Following the application of predefined inclusion and exclusion criteria, 117 individuals with a confirmed diagnosis of Down syndrome were included in the final analysis. Three participants were excluded from the study; two participants were diagnosed with relatively unstable systemic condition and one participant was diagnosed with acute oral infection. Participants were recruited using a non-probability convenience sampling technique from the selected institutions. Enrollment was conducted following coordination with institutional administrators, healthcare providers, and primary caregivers.

### Inclusion and exclusion criteria

#### Inclusion criteria

1. Individuals with a confirmed medical diagnosis of Down syndrome (trisomy 21) documented in institutional records or confirmed by caregiver report with supporting medical documentation.
2. Participants aged between 12 and 25 years inclusive at the time of examination.
3. Individuals actively attending or enrolled in the selected specialized institutions during the study period.
4. Participants for whom written informed consent was obtained from parents or legal guardians prior to any study-related procedures.
5. Individuals capable of cooperating sufficiently to permit a comprehensive clinical oral examination, even if behavioral management techniques or caregiver assistance were required.

#### Exclusion criteria

1. Individuals without a confirmed diagnosis of Down syndrome.
2. Participants with severe, unstable systemic conditions that precluded safe clinical examination (e.g., uncontrolled cardiac disease, severe immunocompromise, active respiratory infection).
3. Individuals with previous orthodontic or orthognathic intervention.
4. Participants who were completely uncooperative and unable to undergo any form of clinical assessment despite behavioral guidance interventions.
5. Individuals whose legal guardians declined participation or withdrew consent at any stage of the study.
6. Patients with acute oral infections or conditions requiring urgent dental treatment that would interfere with comprehensive orthodontic assessment.

The four well trained calibrated examiners; orthodontist and community dentist confirmed testing of the eligible participants against inclusion and exclusion criteria.

The study population included 117 individuals diagnosed with Down syndrome, aged between 12 and 25 years, who were attending the selected institutions during the designated study period. This age range was specifically chosen as it represents a critical developmental window for orthodontic assessment and potential intervention.

### Data collection/variables

Data were systematically collected through comprehensive clinical examinations and structured assessment forms designed specifically for this investigation.

#### Patient history

For each participant, detailed background information was obtained from institutional records and caregiver interviews. This included demographic data (age, sex), comprehensive medical history (systemic health status, regular medication use, history of tonsillectomy), oral hygiene practices (brushing frequency), and presence of relevant oral habits (mouth breathing, night bruxism, ).

#### Clinical examination protocol

Comprehensive clinical assessment included systematic evaluation of the following domains:

- 1. Extra-Oral Facial Analysis: Assessment of facial type (mesocephalic, brachycephalic, dolichocephalic), facial symmetry, lower facial height, lip competence, and upper/lower lip morphology and position.
  Facial Type: The overall shape and structural growth pattern of the face (mesofacial, dolichofacial, or brachyfacial).
  Lower Facial Height: The vertical distance from the base of the nose to the bottom of the chin.
  Lip Morphology: The structural shape, fullness, symmetry, and muscle tone of the lips at rest.
  Such parameters were evaluated on the photographs taken for each individual.
- 2. Profile Facial Analysis: Evaluation of facial profile (convex, straight, concave), nose morphology, nasolabial angle, upper and lower lip positions relative to the esthetic plane, mentolabial sulcus depth, chin position, and mandibular plane angle.
  Nasolabial Angle: The profile angle formed between the base of the nose and the upper lip.
  Mandibular Plane Angle: The steepness of the lower jaw incline relative to the structures of the skull.
  Chin Position: The forward or backward placement of the chin prominence relative to the rest of the face.
- 3. Intra-Oral and Occlusal Findings: Assessment of oral hygiene status using Oral Hygiene Index-Simplified (OHI-S), developed by Greene and Vermillion [14], determined by the sum of two components: the Debris Index and the Calculus Index. In addition, assessment also included dentition type (permanent vs. mixed), molar and canine relationships according to Angle''s classification, anterior overbite; normal (1-3mm), deep(more than 3mm), shallow (less than 1mm), open bite), anterior overjet; normal (2-4mm), excessive (more than 4mm), crossbite, edge-to-edge, and posterior crossbite presence.

The OHI-S index evaluated six specific teeth across six segments of the mouth: buccal surfaces of Maxillary Right First Molar, labial surface of Maxillary Right Central Incisor, buccal surface of Maxillary Left First Molar, lingual surface of Mandibular Left First Molar, labial surface of Mandibular Left Central Incisor and lingual surface of Mandibular Right First Molar.

The OHI-S score is calculated based on surface area coverage:

- Grade 0: No debris, stains or calculus present.
- Grade 1: Soft debris, extrinsic stain or supragingival calculus covering up to 1/3 of the tooth surface.
- Grade 2: Soft debris, extrinsic stain or supragingival calculus covering between 1/3 and 2/3 the tooth surface.
- Grade 3: Soft debris, extrinsic stain or supragingival calculus covering more than 2/3 of the tooth surface.

When the first permanent molars are missing, unerupted, or severely displaced, standard Angle’s classification cannot be directly applied because the primary reference point. Hence, the buccal cusp of the maxillary second premolar is evaluated against the embrasure between the mandibular first and second premolars to determine a molar relationship pattern, as recommended by the American board of Orthodontics.

- 4. Functional and Aesthetic Analysis: Evaluation of respiratory pattern (nasal, oral, both), temporomandibular joint (TMJ) status (normal vs. clicking/pain), smile arc characteristics (normal vs. flat), buccal corridor dimensions (average vs. excessive), and midline shift presence.
  Smile Arc: The relationship between the curve of the upper front teeth and the lower lip when smiling.
  Buccal Corridor: The dark spaces visible at the corners of the mouth between the teeth and lips during a smile.
  Habits were evaluated through caregiver reports and clinical examination.

#### Clinical photography protocol

Standardized intraoral and extraoral photographs were obtained for all cooperative participants. Intraoral photographs were captured using a [Nikon] [model, D5600] digital camera with a [Sigma 105 mm f2.8 macro] macro lens and [Godox mf 12 twinflash K&F, ringflash, Sbarkdent octa v8 difuser], and included the following views: frontal in occlusion, right and left lateral, and maxillary and mandibular occlusal. Cheek retractors and intraoral mirrors were used where tolerated. Extraoral photographs comprised frontal (at rest and smiling), right and left profile views, taken against a neutral background under consistent ambient lighting.

All Photographs were taken in standard settings in manual mode, employing aperture (f/22), shutter speed (1/100s), and low ISO (100) for consistent, deep-focus images. Imaging relies on fixed magnification ratios (1:2.5), and patient positioning in Natural Head Position to eliminate distortion across records.

**Fig. 1:**
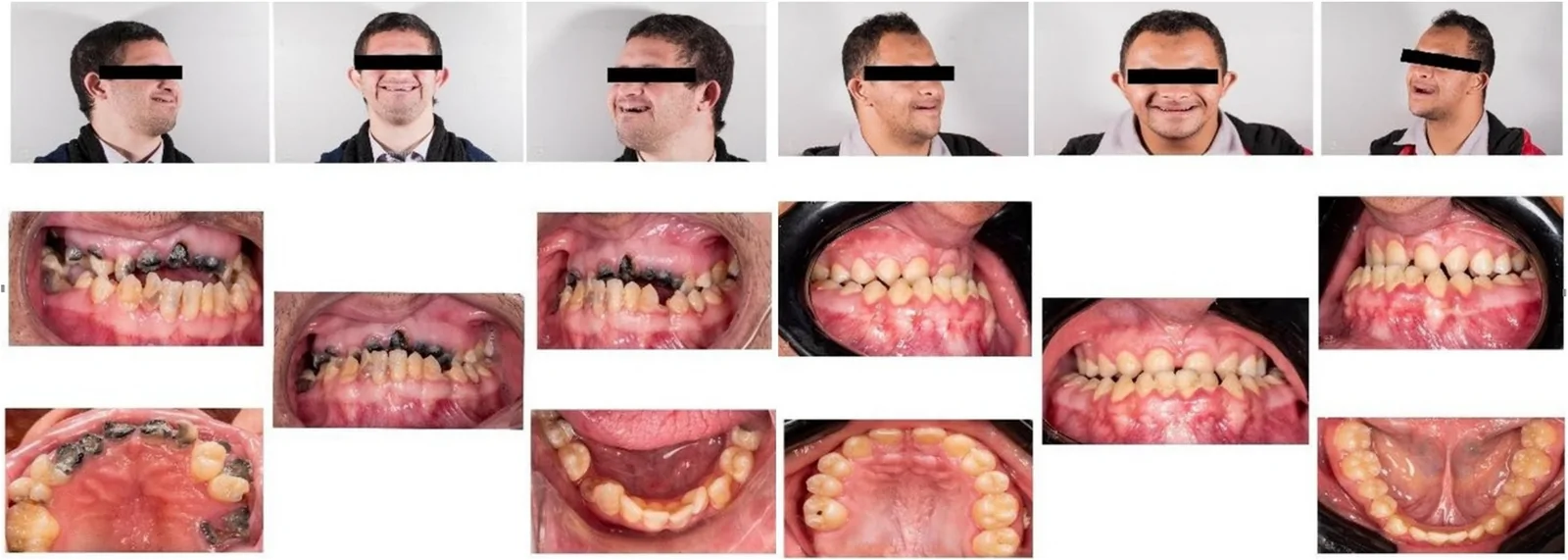
Extra and intra oral photographs for two participants with down syndrome

To safeguard participant anonymity, the ocular region was digitally blurred in all facial photographs. Explicit informed consent for publication of identifiable facial images was obtained. Photographs were acquired only after separate written consent for photography and publication was obtained from parents or legal guardians. Figure 1 illustrates Extraoral and intraoral photographs for two participants with Down Syndrome.

#### Orthodontic treatment need assessment

The Dental Health Component (DHC) of the Index of Orthodontic Treatment Need (IOTN) was utilized to classify orthodontic treatment requirements into five hierarchically ordered grades:

- Grade 1: No treatment need (minor or no occlusal irregularities)
  Ideal occlusion or extremely negligible alignment variations.
- Grade 2: Little treatment need (minor irregularities requiring simple intervention)
  Minor variations including mild rotations, slight crowding under 2mm, or non-traumatic deep bites.
- Grade 3: Borderline need (moderate irregularities where treatment need is equivocal)
  Moderate issues like 3.6–6mm overjets or 2.1–4mm crowding; requires an aesthetic check
- Grade 4: Great treatment need (significant irregularities requiring comprehensive orthodontic intervention)
  High-priority defects including overjets from 6.1–9mm, displacements over 4mm, or traumatic overbites
- Grade 5: Very great treatment need (severe dentofacial discrepancies with profound functional or esthetic impairment)
  Severe anomalies like missing teeth, impacted canines, or overjets exceeding 9mm.

The IOTN-DHC isolates a patient’s single worst occlusal defect by scanning the dentition sequentially through the MOCDO hierarchy (Missing teeth, Overjet, Crossbite, Displacement, Overbite) and recording only the anomaly that yields the highest numerical grade. Any doubtful or borderline cases falling precisely between two categories are systematically resolved by awarding the lower, more conservative treatment grade to prevent over-diagnosis and prioritize public health resources.

Clinical examination and data collection were performed by the four calibrated examiners, both formally trained in the application of the IOTN to ensure consistency, accuracy, and inter/intra examiner reliability.

### Clinical procedures and infection control

**Fig. 2:**
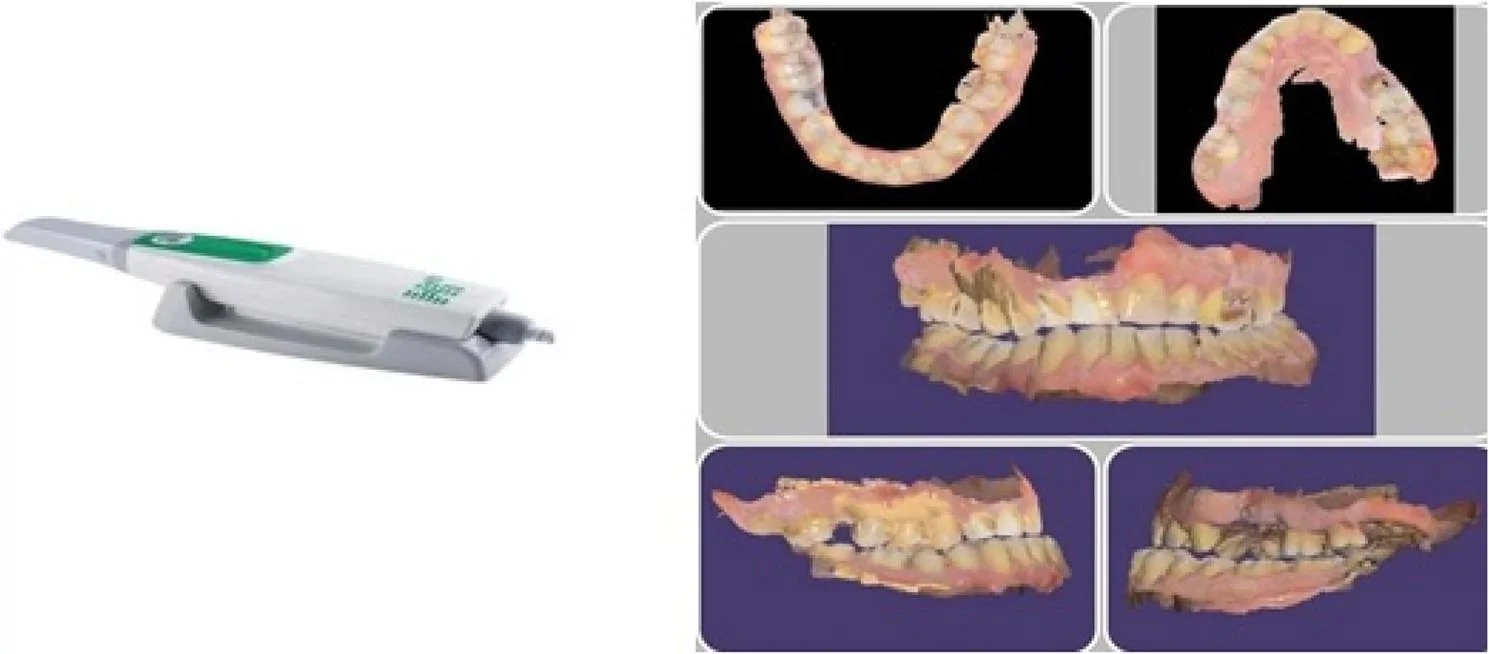
Intra oral scanner used to obtain precise, high-resolution digital dental models

A digital intraoral scanner (Runyes H10, Runyes Medical Instrument Co., Ltd, Ningbo, China) was employed to obtain precise, high-resolution digital impressions of participants’ dentition. Digital scanning offered several advantages in this population, including reduced examination time, elimination of traditional impression material discomfort, and immediate visualization for patient and caregiver education. Figure 2 illustrates the scanner used in the study and the output scans of one of the participants.

Intraoral scanning protocol began at the maxillary right second molar, gliding across the occlusal surfaces to the maxillary left second molar, before rolling to capture the buccal and palatal aspects in a continuous motion. The mandibular arch was scanned next, starting at the mandibular left second molar and finishing at the mandibular right second molar, ensuring full coverage of occlusal, lingual, and buccal boundaries. Finally, a bilateral buccal bite scan was captured in maximum intercuspation from the canine to the first molar regions to digitally articulate the arches.

Digital dental models were generated with incomplete or distorted scans repeated after 2 min interval. The study evaluated occlusal variables, including sagittal overjet, vertical overbite, and specific tooth alignments, molar relationship, all recorded in millimeters (mm) using standardized anatomical landmarks on the incisal edges and molar cusps.

Rigorous infection control protocols were strictly adhered to throughout all phases of the study. Disposable protective barrier sleeves were utilized for the intraoral scanner handpiece and replaced between each participant to prevent cross-contamination. Standard personal protective equipment (PPE), including examination gloves, surgical masks, protective eyewear, and disposable gowns, was worn during all clinical procedures. All non-disposable instruments were sterilized in an autoclave according to established protocols.

**Fig. 3:**
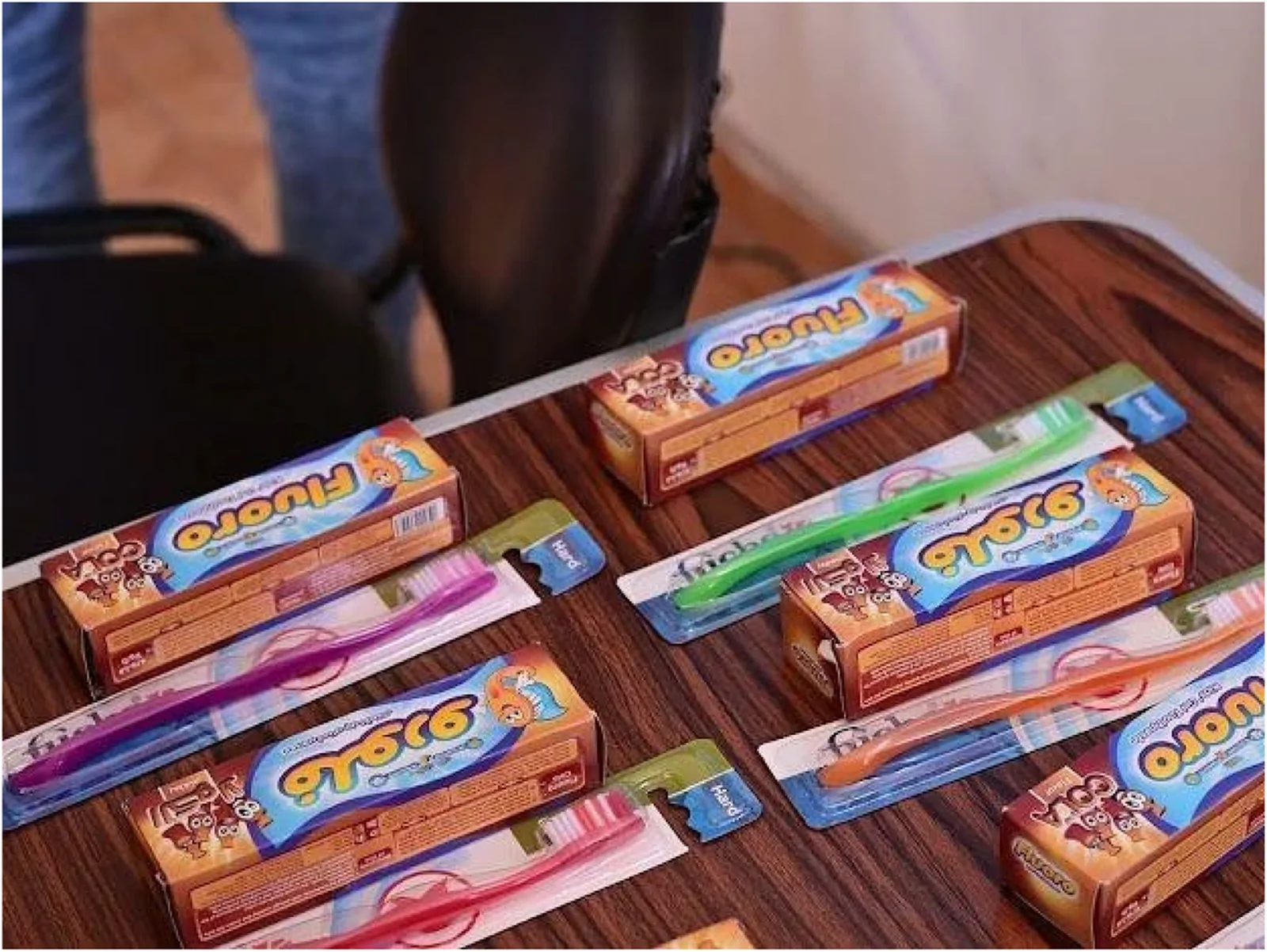
Educational tools utilized as rewards and to demonstrate proper oral hygiene techniques

Educational tools, including visual aids, dental models, and age-appropriate instructional materials, were utilized to demonstrate proper oral hygiene techniques to patients and their caregivers. Oral hygiene instruction was tailored to the cognitive and motor abilities of individual participants. Figure 3 illustrates the educational tools used in the study.

Given the behavioral characteristics commonly associated with Down syndrome, consultation with psychological services and experienced special needs educators was sought to guide appropriate communication strategies and behavior management techniques during examination. Tell-show-do techniques, positive reinforcement, and caregiver presence were employed to maximize cooperation.

Patients identified as requiring primary dental care, including restorative treatment or periodontal therapy (e.g., scaling and root planing), were appropriately referred to affiliated dental clinics or specialist providers prior to or following orthodontic evaluation as clinically indicated.

### Data analysis

All collected data were coded, entered, and analyzed using the Statistical Package for Social Sciences (SPSS) version 21.0 (IBM Corp., Armonk, NY, USA). Descriptive statistics were computed for all study variables. Qualitative (categorical) variables were presented as frequencies (n) and percentages (%). Continuous variables were summarized as mean ± standard deviation (Mean ± SD).

As the primary objective was descriptive, no hypothesis testing was planned or performed; the analysis was limited to descriptive statistics.

### Ethical considerations

This study was conducted in full compliance with the Declaration of Helsinki and the ethical standards of Pharos University. The research protocol was reviewed and approved by the Research Ethics Committee of Pharos University in Alexandria (PUA-REC). Ethical approval was granted on January 6, 2026, with reference approval code PUA-REC-012 (Council Serial: 0220260163463), as illustrated in Appendix 1.

Written informed consent was obtained from the parents or legal guardians of all participants prior to enrollment. The study objectives, examination procedures, potential risks and benefits, and the voluntary nature of participation were thoroughly explained. Caregivers were assured that participation or non-participation would not affect the standard of care provided to their dependents at the participating institutions. Participant confidentiality was strictly maintained through the use of unique identification codes, with all data stored on password-protected, encrypted devices accessible only to the research team. Separate written informed consent was obtained from parents or legal guardians for the acquisition, storage, and potential publication of clinical photographs, with explicit assurance that all identifiable facial images would have the ocular region digitally blurred to preserve anonymity.

## Results

Out of 200 individuals, a total of 117 individuals with Down syndrome were examined and included in the final analysis. Three participants were excluded from the study; two participants were diagnosed with relatively unstable systemic condition and one participant was diagnosed with acute oral infection. The age range of included participants was 12 to 25 years.

### Socio-demographic and behavioral characteristics

As illustrated in Table 1, the study population comprised 117 participants, with a slight female predominance. Specifically, 50 participants (42.7%) were males and 67 participants (57.3%) were females. Regarding age distribution, 47 participants (40.2%) were under 18 years of age, while 70 participants (59.8%), were in the 18–25 years age bracket.

**Table 1:** Socio-demographic and behavioral characteristics (*n* = 117)

Variable	Category	Frequency (*n* = 117)	Percentage (%)
Sex	Male participants	50	42.7%
	Female participants	67	57.3%
Age Group	< 18 years	47	40.2%
	18–25 years	70	59.8%

### Medical and dental history

The majority of participants, 101 individuals (86.3%), were reported to have no additional diagnosed systemic condition, while 16 participants (13.7%) had a documented systemic condition, including controlled cardiac anomalies and thyroid disorders. Regular medication use was reported by 12 participants (10.3%), with the remaining 105 (89.7%) classified as non-users. A history of tonsillectomy was present in 23 participants (19.7%), whereas 94 participants (80.3%) had not undergone the procedure; as shown in Table 2.

**Table 2:** Medical and dental history (*n* = 117)

Variable	Category	Frequency (*n* = 117)	Percentage (%)
Systemic Health	no additional diagnosed systemic condition	101	86.3%
	Systemic Condition (Cardiac)	9	7.7%
	Systemic Condition (thyroid)	7	6%
Regular Medication	Non-User	105	89.7%
	Regular User	12	10.3%
Tonsillectomy	Not Performed	94	80.3%
	Performed	23	19.7%
Oral Habits	Mouth Breathing	38	32.5%
	Night Bruxism	21	17.9%
	No Habits	58	49.6%
Brushing Frequency	Once or twice a day	65	55.6%
	Occasionally/Never	52	44.4%

As shown in Table 2, regarding oral habits, 58 participants (49.6%) reported no deleterious oral habits. Mouth breathing was the most prevalent habit, identified in 38 participants (32.5%), followed by night bruxism in 21 participants (17.9%). Caregiver-reported tooth brushing frequency indicated that 65 participants (55.6%) brushed once or twice daily, while 52 participants (44.4%) brushed occasionally or never.

### Extra-oral facial and profile analysis

As illustrated in Table 3, results revealed that facial type analysis showed a predominance of the mesocephalic pattern, observed in 54 participants (46.2%). Brachycephalic and dolichocephalic facial types were noted in 31 (26.5%) and 32 (27.3%) participants, respectively. Facial symmetry was present in 105 participants (89.7%) exhibiting symmetrical features while only 12 (10.3%) demonstrating asymmetry. Lower facial height was assessed as normal in 58 participants (49.6%), increased in 35 (29.9%), and decreased in 24 (20.5%). Lip competence was documented in 82 participants (70.1%), while incompetence was observed in 35 (29.9%).

**Table 3:** Extra-oral facial analysis (*n* = 117)

Variable	Category	Frequency (*n* = 117)	Percentage (%)
Facial Type	Mesocephalic	54	46.2%
	Brachycephalic	31	26.5%
	Dolichocephalic	32	27.3%
Symmetry	Symmetrical	105	89.7%
	Asymmetrical	12	10.3%
Lower Facial Height	Normal	58	49.6%
	Increased	35	29.9%
	Decreased	24	20.5%
Lip Competence	Competent	82	70.1%
	Incompetent	35	29.9%
Upper Lip	Average	76	65.0%
	Short	29	24.8%
	Long	12	10.3%
Lower Lip	Average	88	75.2%
	Everted	18	15.4%
	Tense	11	9.4%

Profile facial analysis demonstrated a convex facial profile in 58 participants (49.6%), a straight profile in 28 (23.9%), and a concave profile in 31 (26.5%). Nose morphology was classified as normal in 85 participants (72.6%), upturned in 12 (10.3%), and downturned in 20 (17.1%). Nasolabial angle assessment revealed normal angulation in 60 participants (51.3%), acute angulation in 32 (27.3%), and obtuse angulation in 25 (21.4%). Chin position was normal in the majority of participants (85, 72.6%), while prognathic and retrognathic chin positions were noted in 20 (17.1%) and 12 (10.3%) participants, respectively; as shown in Table 4.

**Table 4:** Profile facial analysis (*n* = 117)

Variable	Category	Frequency (*n* = 117)	Percentage (%)
Facial Profile	Convex	58	49.6%
	Straight	28	23.9%
	Concave	31	26.5%
Nose Morphology	Normal	85	72.6%
	Upturned	12	10.3%
	Downturned	20	17.1%
Naso-Labial Angle	Normal(90–120°)	60	51.3%
	Acute (< 90°)	32	27.3%
	Obtuse (> 120°)	25	21.4%
Upper Lip Position	Normal	74	63.3%
	Protruded	28	23.9%
	Retruded	15	12.8%
Lower Lip Position	Normal	70	59.8%
	Protruded	31	26.5%
	Retruded	16	13.7%
Mento-labial Sulcus	Normal	65	55.6%
	Deep	32	27.3%
	Flat	20	17.1%
Chin Position	Normal	85	72.6%
	Prognathic	20	17.1%
	Retrognathic	12	10.3%
Mandibular Plane	Normal	70	59.8%
	Steep	35	29.9%
	Flat	12	10.3%

### Intra-oral and occlusal findings

Oral hygiene index- simplified was rated as poor (3.1-6) in 55 participants (47.0%), fair (1.3-3) in 40 (34.2%), and good (0-1.2) in 22 (18.8%). The majority of participants (95, 81.2%) presented with permanent dentition, while 22 (18.8%) were in the mixed dentition phase; as illustrated in Table 5.

**Table 5:** Intra-oral and occlusal findings (*n* = 117)

Variable	Category	Frequency (*n* = 117)	Percentage (%)	95% Confidence Interval
Oral Hygiene Index Simplified	0–1.2	22	18.8%	12.8%–26.8%
	1.3–3	40	34.2%	26.2%–43.2%
	3.1–6	55	47.0%	38.2%–56.0%
Dentition Type	Permanent	95	81.2%	73.2%–87.2%
	Mixed (Early/Late)	22	18.8%	12.8%–26.8%
Molar Relation	Class I	48	41.0%	32.5%–50.1%
	Class II	35	29.9%	22.4%–38.7%
	Class III	34	29.1%	21.6%–37.8%
Canine Relation	Class I	50	42.7%	34.1%–51.8%
	Class II	36	30.8%	23.1%–39.6%
	Class III	31	26.5%	19.3%–35.1%
Anterior Overbite	Normal	62	53.0%	44.0%–61.8%
	Deep bite	9	7.7%	2.7%–12.7%
	Shallow bite	19	16.2%	11.2%-21.2%
	Open bite	27	23.1%	16.4%–31.5%
Anterior Overjet	Normal	55	47.0%	38.2%–56.0%
	Excessive	33	28.2%	20.8%–37.0%
	Crossbite	23	19.7%	14.7%–24.7%
	Edge to Edge	6	5.1%	1.1%-10.1%
Posterior Crossbite	Absent	92	78.6%	70.4%–85.1%
	Present	25	21.4%	14.9%–29.6%

Molar relationship according to Angle’s classification revealed Class I molar relationship in 48 participants (41.0%), Class II in 35 (29.9%), and Class III in 34 (29.1%). Canine relationship was Class I in 50 participants (42.7%), Class II in 36 (30.8%), and Class III in 31 (26.5%). Anterior overbite was within normal limits in 62 participants (53.0%), with deep or shallow bite in 28 (23.9%) and anterior open bite in 27 (23.1%). Anterior overjet was normal in 55 participants (47.0%), excessive in 33 (28.2%), and presented as anterior crossbite or edge-to-edge relationship in 29 (24.8%). Posterior crossbite was present in 25 participants (21.4%); as illustrated in Table 5.

### Functional, aesthetic, and smile analysis

Respiration pattern was predominantly nasal in 74 participants (63.3%), oral in 28 (23.9%), and mixed in 15 (12.8%). Temporomandibular joint (TMJ) status was normal in 90 participants (76.9%), while clicking sounds or pain upon palpation were elicited in 27 participants (23.1%). Smile arc analysis demonstrated a normal curvature in 79 participants (67.5%) and a flat smile arc in 38 (32.5%). Buccal corridor space was average in 84 participants (71.8%) and excessive in 33 (28.2%). Midline shift was absent in 76 participants (65.0%) and present (either maxillary, mandibular, or both) in 41 participants (35.0%); as illustrated in Table 6.

**Table 6:** Functional, aesthetic, and smile analysis (*n* = 117)

Variable	Category	Frequency (*n* = 117)	Percentage (%)
Respiration	Nasal	74	63.3%
	Oral	28	23.9%
	Mixed nasal and oral breathing	15	12.8%
TMJ Status	Normal	90	76.9%
	Clicking	21	18%
	Pain	6	5.1%
Smile Arc	Normal	79	67.5%
	Flat	38	32.5%
Buccal Corridor	Average	84	71.8%
	Excessive	33	28.2%
Midline Shift	Absent	76	65.0%
	Present (Upper)	17	14.5%
	Present (Lower)	24	20%

### Orthodontic Treatment Need (IOTN Assessment)

Assessment of orthodontic treatment need using the Dental Health Component (DHC) of the Index of Orthodontic Treatment Need (IOTN) revealed that Grade 4 (great treatment need) was the most prevalent category, encompassing 39 participants (33.3%). This was followed by Grade 3 (borderline need) in 26 participants (22.2%) and Grade 5 (very great treatment need) in 19 participants (16.2%). Grades 1 and 2, indicating no or little treatment need, were observed in 12 (10.3%) and 21 (17.9%) participants, respectively; as illustrated in Table 7.

**Table 7:** Distribution of IOTN grades (*n* = 117)

IOTN-DHC grade	Classification	Frequency (*n* = 117)	Percentage (%)	95% Confidence Interval
Grade 1	No treatment need	12	10.3%	6.0% – 17.1%
Grade 2	Little treatment need	21	17.9%	12.0% – 25.9%
Grade 3	Borderline treatment need	26	22.2%	15.6% – 30.6%
Grade 4	Great treatment need	39	33.3%	25.4% – 42.3%
Grade 5	Very great treatment need	19	16.2%	10.6% – 24.0%
Total		117	100%	

Cumulatively, 49.6% of the study population (*n* = 58) demonstrated a definitive need for orthodontic intervention (Grades 4–5)

## Discussion

The findings of the present investigation provide a comprehensive characterization of malocclusion patterns, associated craniofacial features, and orthodontic treatment needs among a cohort of 117 individuals with Down syndrome. The results unequivocally indicate a substantial burden of malocclusion and a pronounced need for orthodontic intervention, with approximately half of the participants (49.6%) exhibiting great to very great treatment requirements according to the IOTN.

### Orthodontic treatment need and malocclusion patterns

The observed prevalence of definitive or borderline orthodontic treatment need (Grades 4–5: 49.6%) is consistent with previously reported figures in the international literature. However, in a cross-sectional study of individuals with Down syndrome, Alkawari documented that 81.9% required orthodontic treatment based on IOTN assessment [13]. The slight discrepancy between these figures may be attributable to variations in sample size, age distribution, geographic and ethnic differences in craniofacial morphology, or subtle differences in examiner calibration. Nevertheless, both studies converge on the overarching conclusion that the vast majority of individuals with Down syndrome present with malocclusions of sufficient severity to warrant professional orthodontic evaluation and, in many cases, intervention.

Of particular clinical significance is the finding that 49.6% of participants were categorized as IOTN Grades 4 or 5, indicating great or very great treatment need. These grades correspond to severe occlusal discrepancies including, but not limited to, increased overjet greater than 9 mm, reverse overjet greater than 3.5 mm with reported masticatory or speech difficulties, anterior or posterior crossbites with associated mandibular displacement, severe open bites, and hypodontia requiring complex interdisciplinary management. The high proportion of patients falling into these severe categories underscores the critical importance of early identification and referral to specialist orthodontic services equipped to manage complex cases in the context of intellectual and developmental disabilities.

Regarding Angle’s classification, the distribution of molar relationships in our cohort—Class I (41.0%), Class II (29.9%), and Class III (29.1%)—differs somewhat from the pattern reported by Alkawari [13], who identified Class III malocclusion as the predominant pattern (59.1%) in a relatively smaller sample (23 children aged 10–14 years). While Class III malocclusion is classically associated with Down syndrome due to maxillary hypoplasia and relative mandibular prognathism, our findings suggest a more heterogeneous distribution. Alternatively, this finding may indicate phenotypic variability within the Down syndrome population. Longitudinal follow-up of this cohort would be valuable to assess whether the prevalence of Class III malocclusion increases with age.

The prevalence of anterior open bite and posterior crossbite in our sample confirms previously reported studies on Down syndrome populations [6, 7]. These occlusal features are multifactorial in etiology, resulting from the interplay of skeletal maxillary transverse deficiency and mouth breathing. Mouth breathing was observed in 32.5% of participants, while oral respiration, either alone or combined with other breathing patterns, was found in 36.7%. These findings further support the theory possibly attributing the development of vertical and transverse occlusal problems in this population to the altered breathing patterns in the.

### Oral health status and behavioral considerations

The oral hygiene in this study warrant particular attention. Only 18.8% of participants demonstrated good oral hygiene, while 81.2% were rated as having fair or poor oral hygiene. These findings, while not unexpected given the well-documented challenges of maintaining optimal oral hygiene in individuals with intellectual disabilities, highlight a critical area for intervention [15]. The high proportion of participants reporting infrequent brushing (44.4% brushing occasionally or never) underscores the need for targeted oral health education and support for both patients and caregivers [16]. However, toothbrushing frequency was apparently reported by participants or their caregivers, which may have introduced recall and social desirability bias. This might be considered as a possible limitation of our study.

### Clinical implications and management considerations

The management of malocclusion in patients with Down syndrome may require a slightly different approach from conventional orthodontic treatment philosophies. The goal of treatment should not be solely defined by the achievement of ideal occlusal relationships as described by Angle, but rather by the clinician’s ability to balance biomechanical principles with the biological, behavioral, and medical realities of this population. Key considerations include:

- Biomechanical Modifications: The application of light, continuous, and controlled orthodontic forces is paramount in patients with Down syndrome to minimize any prevalence of periodontal compromise, and reduce any potential for root resorption. Treatment mechanics should be simplified where possible, and treatment duration should be realistic, acknowledging that extended treatment times may be necessary.
- Periodontal Considerations: Only 18.8% of participants had good oral hygiene, while 81.2% had fair or poor oral hygiene. These findings are not surprising, considering the known difficulties in maintaining good oral hygiene among individuals with Down syndrome. Given the elevated risk of periodontal disease in Down syndrome, meticulous attention to oral hygiene throughout orthodontic treatment is non- negotiable [17]. Pre-orthodontic periodontal stabilization, including professional prophylaxis and oral hygiene instruction, is essential.
- Behavioral and Communication Strategies: Successful orthodontic treatment in this population depends on building trust with patients and caregivers. Appointments should allow enough time, with simple and clear communication in a calm and predictable environment. Support from a multidisciplinary team can also improve the treatment experience and outcomes.

### Comparison with international literature

The findings of this study align with a growing body of international literature documenting the elevated orthodontic needs of individuals with Down syndrome. In contrast to the findings of our study, a Study by AlSarheed reported high IOTN grades and a predominance of Class III malocclusion (45% − 50%), anterior open bite, and posterior crossbite [18]. In contrast to previous study by Oredugba addressing the oral prophylaxis need and restorative clinical treatment need, the present investigation adds valuable data, highlighting orthodontic treatment need in individuals with Down syndrome [19].

### Study strengths

This study possesses several notable strengths. First, the relatively large sample size (*n* = 117) compared to previous studies. Second, the comprehensive nature of the clinical assessment, encompassing extra-oral facial analysis, profile evaluation, intra-oral findings, functional parameters, and aesthetic considerations, provides a better characterization of the Down syndrome craniofacial and occlusal phenotype. Third, the use of calibrated examiners and a validated, internationally recognized index (IOTN) ensure methodological rigor and facilitates comparison with other studies. Fourth, the incorporation of digital intraoral scanning represents a contemporary approach to data collection in an attempt to minimize patient discomfort and provides a permanent, high-resolution record for future reference and analysis.

## Study limitations

Despite the contributions of this investigation, several limitations must be acknowledged when interpreting the findings.

First, the study employed a non-probability convenience sampling technique, which may introduce selection bias. Participants were recruited exclusively from specialized institutions, and the findings may not be fully generalizable to individuals with Down syndrome residing in other geographic regions, those attending mainstream educational settings, or those not engaged with institutional care. Possible clustering might have added power to the current study.

Second, the cross-sectional design precludes the assessment of longitudinal changes in occlusal relationships and treatment needs over time.

Third, while the IOTN Dental Health Component is a validated and widely used index, it does not capture the full spectrum of factors influencing orthodontic treatment decisions, particularly the Aesthetic Component, patient/parent perceptions, and psychosocial considerations. The inclusion of the Aesthetic Component and quality-of-life measures in future studies would provide a more comprehensive assessment of treatment need. Moreover, Dental Health Component assesses normative need and does not necessarily reflect treatment demand, feasibility, patient preference or likely benefit.

Fourth, patient cooperation, while manageable for examination purposes in the majority of cases, posed challenges in a subset of participants (24.8% uncooperative). This may have influenced the accuracy and completeness of certain clinical assessments, particularly those requiring sustained patient compliance (e.g., intraoral scanning, detailed occlusal evaluation). Excluding individuals who could not cooperate may produce selection bias and underestimate the clinical challenges and treatment needs of individuals with more severe disability.

Fifth, the study did not include radiographic assessment (e.g., panoramic or cephalometric radiographs). Consequently, the presence of impacted teeth, congenitally missing teeth, root morphology anomalies, and precise skeletal relationships could not be fully characterized. Despite the ethical concerns regarding patient exposure to radiation, radiographic evaluation would enhance diagnostic precision and inform comprehensive treatment planning. The lack of imaging prevented assessment of skeletal relationships and dental anomalies.

Another possible limitation in the current study is the potential misclassification. Moreover, oral habits, medical history and brushing frequency appear to rely partly on caregiver reports and may be affected by recall bias. In addition, The cross-sectional design does not permit causal conclusions regarding relationships between habits, breathing patterns, oral hygiene and malocclusion. Finally, No control group of individuals without Down syndrome was included. Therefore, the study cannot determine whether the observed prevalence differs from that in the general population.

## Conclusion

In this institution-based sample of individuals with Down syndrome, 22.2% had borderline orthodontic treatment need (IOTN-DHC Grade 3), while 49.6% had great or very great treatment need (Grades 4–5). Class I was the most frequent molar relationship, although Class II and Class III relationships were also common. Anterior open bite, posterior crossbite and suboptimal oral hygiene were frequently observed. These findings support the importance of accessible orthodontic assessment and preventive oral-health support for individuals with Down syndrome. However, the convenience sampling method limits generalization beyond the participating institutions.

## Recommendations

### Recommendations for clinical practice

Based on the findings of this study, the following recommendations are proposed for clinical practice:

1. Early Orthodontic Screening: Orthodontic screening is recommended for individuals with Down syndrome by 7–8 years of age, or earlier when significant occlusal abnormalities are identified, to facilitate timely intervention and referral.
2. Multidisciplinary Care Coordination: Clinical management of individuals with Down syndrome should incorporate a multidisciplinary approach, with coordination among dental and medical professionals and relevant allied health specialists to address the patient’s comprehensive functional and health needs.
3. Individualized Treatment Planning: Orthodontic treatment should be individualized according to the patient’s medical and behavioral status, oral hygiene capacity, and caregiver support. Treatment priorities should emphasize functional improvement, oral hygiene facilitation, and facial esthetics rather than strict achievement of ideal occlusal norms.
4. Enhanced Oral Hygiene Support: Given the high prevalence of fair/poor oral hygiene in this population, intensive preventive education and support for patients and caregivers must be a cornerstone of orthodontic management. Simplified oral hygiene regimens, the use of adjunctive aids (e.g., electric toothbrushes, floss holders), and regular professional maintenance are essential.

### Recommendations for future research

Future investigations should address the limitations of the present study and expand upon its findings. Specific recommendations include:

1. Larger, multi-centre Studies: Future research should aim for larger, more representative samples drawn from diverse geographic regions and care settings across Egypt to enhance generalizability.
2. Longitudinal Cohort Studies: Prospective, longitudinal studies tracking occlusal changes and orthodontic treatment outcomes over time are needed to understand the natural history of malocclusion in Down syndrome and to evaluate the long-term stability of orthodontic interventions.
3. Inclusion of Radiographic and Cephalometric Analysis: Future studies should incorporate radiographic imaging (panoramic and lateral cephalometric radiographs) to comprehensively characterize dental anomalies (hypodontia, impactions) and skeletal relationships, thereby enabling more precise diagnostic classification and treatment planning. However, future radiographic studies should still follow clinical-justification and radiation-protection principles.
4. Assessment of Quality of Life and Patient-Reported Outcomes: The incorporation of validated oral health-related quality-of-life (OHRQoL) instruments and caregiver perception surveys would provide valuable insights into the functional and psychosocial impact of malocclusion and the perceived benefits of orthodontic treatment from the patient and family perspective.
5. Intervention Studies: Well-designed initial prospective feasibility studies evaluating the efficacy, safety, and feasibility of different orthodontic treatment modalities (e.g., conventional fixed appliances vs. clear aligners vs. simplified removable appliances) in the Down syndrome population are recommended to guide evidence-based clinical decision-making.

## Supplementary Information

Supplementary Material 1: Appendix 1. Ethical Approval.

Supplementary Material 2.

Supplementary Material 3.

Supplementary Material 4.

Supplementary Material 5.

Supplementary Material 6.

Supplementary Material 7.

## Data Availability

The datasets analyzed during the current study are available from the corresponding author upon reasonable request.

## Abbreviations

IOTN: Index of Orthodontic Treatment Need
DS: Down Syndrome
DHC: Dental Health Component
OHI-S: Oral Hygiene Index-Simplified
TMJ: Temporomandibular Joint
PPE: Personal protective equipment
SPSS: Statistical Package for Social Sciences
OHRQoL: Oral Health-Related Quality-Of-Life
