# Supplementary material for: Malocclusion and orthodontic treatment needs among individuals with down syndrome in Egypt: a multicentre cross-sectional study
Source: BMC Oral Health. 2026 Oct 19;26:2060. doi: 10.1186/s12903-026-09995-9 (PMC13679113; doi:10.1186/s12903-026-09995-9)
Supplement: Supplementary file 1 [file 12903_2026_9995_MOESM1_ESM.pdf]

PHAROS UNIVERSITY IN ALEXANDRIA
Research Ethics Committee

جـامعــة فــاروس بالإســكندريــة
لجنة أخلاقيات البحث العلمي

Review Report

(PUA-REC-012)

PUA	Code of Faculty	yy	mm	dd	MS (1) – MD (2) – Research (3) – Project (4)	Serial
	02	2026	01	6	3	463

Date of PUA-REC council: 6 / 1 / 2026

Type of research: **Promotion Research**

Title of research: **Assessment of malocclusion patterns and orthodontic treatment needs in children with Down syndrome**

Provided By

Dr. Hani AlSwafeeri
Department of Orthodontics, Faculty of Dentistr, Pharos University in Alexandria, Alexandria, Egypty

Student. Hala Ahmed
BDS of Dentistry, Faculty of Dentistry, Pharos University in Alexandria, Alexandria, Egypt

Student. Mariam Saaied Badran
BDS of Dentistry, Faculty of Dentistry, Pharos University in Alexandria, Alexandria, Egypt

Student. Menna Allah Mohamed
BDS of Dentistry, Faculty of Dentistry, Pharos University in Alexandria, Alexandria, Egypt

Student. Nada Shehata
BDS of Dentistry, Faculty of Dentistry, Pharos University in Alexandria, Alexandria, Egypty

Dr. Nancy Bedweni
Dental Public Health and Preventive Dentistry Department, Faculty of Dentistry, Pharos University in Alexandria, Alexandria, Egypty

Reviewer report:

✓ Accepted according to ethical standards of scientific research
☐ Accepted after correction
☐ Not accepted
☐ Other decision

Head of Research Ethics Committee
Name: Prof. Rasha Shaalan
6/ 1 / 2026
Rshad

Pharos University in Alexandria
PUA-REC
PUA-Research Ethics Committee

Address: Pharos University, Canal El Mahmoudia Street, beside Green Plaza, Alexandria Governorate, 21648
Website: https://www.pua.edu.eg **Tel.:** +(203) 3877700
E-mail: ureac@pua.edu.eg
