# Supplementary material for: Malocclusion and orthodontic treatment needs among individuals with down syndrome in Egypt: a multicentre cross-sectional study
Source: BMC Oral Health. 2026 Oct 19;26:2060. doi: 10.1186/s12903-026-09995-9 (PMC13679113; doi:10.1186/s12903-026-09995-9)
Supplement: Supplementary file 2 [file 12903_2026_9995_MOESM2_ESM.docx]

**استمارة الموافقة المستنيرة**

(PUA-REC-004)

| **اسم المشارك** | مريم سعيد بدران  ندي زكريا شحاته  حلا أحمد سمير  منه الله محمد حسن |
| --- | --- |
| **عنوان البحث** | **Assessment of malocclusion patterns and orthodontic treatment needs in children with Down syndrome** |
| **الباحث الرئيسي** | **Hani AlSwafeeri** |
| **تليفون الباحث الرئيسي** | **01001830264** |

## سيشرح لك عضو من فريق البحث محتويات هذه الدراسة وتأثيرها عليك. و يصف هذا الإقرار إجراءات الدراسة ، والمخاطر والفوائد من المشاركة ، وكيفية الحفاظ على سرية المعلومات. وهذه الموافقة تسمى الموافقة المستنيرة. إذا قررت المشاركة في هذه الدراسة، سيطلب منك التوقيع على هذا الإقرار.

**معلومات عن البحث تهم المشاركين بالدراسة:**

| **لماذا تجري هذه الدراسة؟** | **للتحقق عن مدي احتياج أطفال ذو متبازم** |
| --- | --- |
| **كم عدد المشاركين في هذه الدراسة ؟** | **117** |
| **ما هي الاجراءات المتبعة في الدراسة؟** | **بتحليل مقاسات الاسنان الخاصة بهم على برنامج** |
| **مكان البحث** | **جامعة فاروس** |
| **ما هي مسئوليات المبحوثين المشاركين بالدراسة؟** | **لا ينطبق** |
| **ما هي مدة مشاركة المبحوثين المشاركين بالدراسة؟** | **لا توجد مدة محددة نظرا لان الدراسة تقوم بتحليل مقاسات الاسنان الخاصة بهم** |
| **هل يستطيع المبحوثين المشاركين بالدراسة الانسحاب من الدراسة بأمان قبل موعد انتهاء الدراسة؟** | **نعم** |
| **هل هناك مخاطر متوقعة للمبحوثين بعد الانتهاء من الدراسة؟** | **لا** |
| **ما هي المخاطر أو الآثار الجانبية التي يمكن حدوثها للمبحوثين من جراء المشاركة في هذه الدراسة ؟** | **لا** |
| **هل هناك فوائد للمبحوثين نتيجة المشاركة في هذه الدراسة ؟** | **لا** |
| **ما الاجراء إذا تعرض المبحوثين لإصابة نتيجة المشاركة في الدراسة؟** | **لا توجد اى مخاطر للاصابات** |
| **هل سيتحمل المبحوثين أي تكاليف؟** | **لا** |
| **هل سيتقاضى المبحوثين أجر نظير الاشتراك في الدراسة؟ برجاء تحديد المبلغ ان وجد** | **لا** |
| **هل سيتم الحفاظ علي سرية المعلومات الطبية الخاصة بالمبحوثين؟** | **نعم** |
| **ما هي حقوق المبحوثين إذا وافقوا على المشاركة في هذه الدراسة ؟** | **سرية البيانات الخاصة بهم وعدم وجود اي ضرر بهم.** |
| **هل يمكن للمبحوثين الاتصال إذا كانت لديهم أي أسئلة أو مشاكل؟** | **نعم** |

**إقرار بالموافقة**

**المشارك:**

لقد تم شرح البحث والإجراءات لي. وسمح ي بأن اسأل أي سؤال لدي في هذا الوقت. ويمكنني أن اسأل أي أسئلة إضافية في وقت لاحق. ويمكنني إنهاء المشاركة في الدراسة في أي وقت دون يؤثر ذلك علي شيء.

سأحصل علي نسخة موقعة من هذا الإقرار بالموافقة.

أنا اقر بالموافقة على المشاركة في هذه الدراسة. موافقتي طوعية, ولست بحاجة إلى التوقيع على هذا الإقرار إذا كنت لا أريد المشاركة في هذه الدراسة البحثية.

أوافق على أن الباحثين في هذه الدراسة والمتعاونين معهم سوف يستخدمون العينات الخاصة بي في هذه الدراسة ودراسات أخرى إذا لزم الأمر

توقيع المشارك:

التاريخ

**الباحث الرئيسي:**

لقد شرحت طبيعة الدراسة والغرض منها وما تنطوي عليه من مخاطر. وقد أجبت وسأجيب علي الأسئلة على أفضل قدر من استطاعتي. سأعطي نسخة موقعة من الإقرار بالموافقة إلي المشارك المذكور أعلاه.

**توقيع الباحث الرئيسي**

**د. هانى السوافيرى**

الشخص المعني بالدراسة غير قادر على الموافقة بنفسه لأنه / إنها قاصر. من خلال التوقيع أدناه، أنت تعطي إذنك لطفلك بان يضمن في هذه الدراسة

**الأبوين أو الوصي قانونا-----------------**

التاريخ: /	/
