# Supplementary material for: Malocclusion and orthodontic treatment needs among individuals with down syndrome in Egypt: a multicentre cross-sectional study
Source: BMC Oral Health. 2026 Oct 19;26:2060. doi: 10.1186/s12903-026-09995-9 (PMC13679113; doi:10.1186/s12903-026-09995-9)
Supplement: Supplementary file 3 [file 12903_2026_9995_MOESM3_ESM.docx]

**Study protocol**

| **Hani AlSwafeeri** | **Principle investigator** |
| --- | --- |
| **Assessment of malocclusion patterns and orthodontic treatment needs in children with Down syndrome** | **Study title** |

**Study background (not more than half a page)**

Down syndrome (DS) is a genetic disorder often associated with distinctive craniofacial features that predispose affected children to various malocclusion patterns and Epidemiologic and systematic reviews show that individuals with Down syndrome have a substantially higher prevalence of malocclusion (especially Angle Class III and crossbites) than the general population. Meta-analytic data and cross-sectional studies frequently report Class III tendency, anterior and posterior crossbites, and anterior open bite as common findings; malocclusion prevalence in many series exceeds that of age-matched peers.

Dental developmental differences commonly seen in Down syndrome—delayed eruption, hypodontia, short tooth roots, enamel defects, and higher prevalence of periodontal disease—affect timing, feasibility and prognosis of orthodontic interventions and increase the need for multidisciplinary planning (orthodontics, pediatric dentistry, periodontics, ENT, speech/feeding therapists). Behavioral issues and medical comorbidities (cardiac, respiratory, immunologic) further influence treatment planning and risk assessment.

Indexes such as the Index of Orthodontic Treatment Need (IOTN) have been used to quantify treatment necessity in special-needs populations; studies applying IOTN in groups of children with Down syndrome report higher levels of treatment need compared with non-syndromic peers, emphasizing an unmet demand for orthodontic care in many settings.

Despite the well-documented pattern of craniofacial differences and malocclusion, there remain geographic and service-level gaps in the evidence: prevalence estimates vary by age group and methodology, and data on local treatment needs, barriers to care, and outcomes of early vs. delayed orthodontic intervention are limited in many regions. This justifies focused cross-sectional assessments that (1) describe malocclusion patterns in defined pediatric Down syndrome cohorts, (2) quantify treatment need using standardized indices (e.g., IOTN), and (3) identify practical considerations for local service planning and multidisciplinary management.

we will be

Assessing and evaluating IOTN , photographing 117 patients with Down syndrome extraorally and intraorally and orthodontic treatment needs for Down syndrome patients

**Study Aim**

**Asses the prevalence of various types of malocclusion in children with down Syndrome, Evaluate the orthodontics needs using the index of orthodontic treatment ( IOTN)**

**Study methods (not more than one page)**

117 children will be intraoral scanned and will be collected and photographed , Evaluate the orthodontic needs using the ( IOTN)

Principal Investigator	Name: Hani Alswafeeri. Signature: 13/10/2025
