# Supplementary material for: Malocclusion and orthodontic treatment needs among individuals with down syndrome in Egypt: a multicentre cross-sectional study
Source: BMC Oral Health. 2026 Oct 19;26:2060. doi: 10.1186/s12903-026-09995-9 (PMC13679113; doi:10.1186/s12903-026-09995-9)
Supplement: Supplementary file 4 [file 12903_2026_9995_MOESM4_ESM.docx]

**Application Checklist (PUA-REC-005)**

**نموذج قائمة تدقيق**

**عنوان البحث:** **Assessment of malocclusion patterns and orthodontic treatment needs in children with Down syndrome**

**للأبحاث البشرية الطبية:**

| نعم | لا | لا ينطبق | قائمة تدقيق | ملاحظات |
| --- | --- | --- | --- | --- |
| **☒** | **☐** | **☐** | "نموذج مقترح البحث" : هل النموذج كامل وموقع ومؤرخ من الباحث الرئيسي؟ |  |
| **☒** | **☐** | **☐** | هل الاعتبارات الأخلاقية الموافق عليها الباحث تتطابق مع وصف الدراسة |  |
| **☐** | **☐** | **☒** | إذا كانت الدراسة تجربة إكلينيكية، يجب إرفاق النماذج المخصصة للموافقة المستنيرة للتجارب الإكلينيكية بعد إكمالها وتوقيعها |  |
| **☒** | **☐** | **☐** | هل الهدف العلمي من البحث يبرر إجراءه على البشر؟ |  |
| **☒** | **☐** | **☐** | هل تم تبرير الجدارة العلمية للتعامل مع عينة الدراسة؟ |  |
| **☒** | **☐** | **☐** | هل نوع وعدد عينة الدراسة مناسب لنوع البحث؟ |  |
| **☐** | **☐** | **☒** | هل من الضرورة مشاركة الفئات المستحقة للحماية الإضافية في البحث؟ |  |
| **☒** | **☐** | **☐** | هل ضمانات حماية الفئات المستحقة كافية؟ |  |
| **☐** | **☐** | **☒** | هل طرق تخفيف المخاطر كافية؟ |  |
| **☒** | **☐** | **☐** | هل طرق وإجراءات حماية سرية وخصوصية البيانات كافية؟ |  |
| **☐** | **☒** | **☐** | هل يؤثر تضارب المصالح على المشاركين أو نتائج البحث؟ |  |

**اسم المراجع/المراجعبن	رئيس اللجنة**

**التوقيع**

**التاريخ**

**للأبحاث البشرية غير الطبية:**

| نعم | لا | لا ينطبق | قائمة تدقيق | ملاحظات |
| --- | --- | --- | --- | --- |
| **☐✅** | **☐** | **☐** | "نموذج مقترح البحث" : هل النموذج كامل وموقع ومؤرخ من الباحث الرئيسي؟ |  |
| **✅☐** | **☐** | **☐** | هل الاعتبارات الأخلاقية الموافق عليها الباحث تتطابق مع وصف الدراسة |  |
| **☐** | **✅☐** | **☐** | هل يتضمن البحث جمع معلومات حساسة ثقافيًا أو مغايرة عن التقاليد الراسخة. |  |
| **☐✅** | **☐** | **☐** | هل الهدف العلمي من البحث يبرر إجراءه على البشر؟ |  |
| **☐✅** | **☐** | **☐** | هل تم تبرير الجدارة العلمية للتعامل مع عينة الدراسة؟ |  |
| **✅☐** | **☐** | **☐** | هل نوع وعدد عينة الدراسة مناسب لنوع البحث؟ |  |
| **✅☐** | **☐** | **☐** | هل من الضرورة مشاركة الفئات المستحقة للحماية الإضافية في البحث؟ |  |
| **☐✅** | **☐** | **☐** | هل ضمانات حماية الفئات المستحقة كافية؟ |  |
| **☐✅** | **☐** | **☐** | هل طرق تخفيف المخاطر كافية؟ |  |
| **☐✅** | **☐** | **☐** | هل طرق وإجراءات حماية سرية وخصوصية البيانات كافية؟ |  |
| **☐** | **☐✅** | **☐** | هل يؤثر تضارب المصالح على المشاركين أو نتائج البحث؟ |  |

**اسم المراجع/المراجعبن	رئيس اللجنة**

**التوقيع**

**التاريخ**

**للأبحاث على الحيوانات:**

| نعم | لا | لا ينطبق | قائمة تدقيق | ملاحظات |
| --- | --- | --- | --- | --- |
| **☐** | **☐** | **☐** | "نموذج مقترح البحث" : هل النموذج كامل وموقع ومؤرخ من الباحث الرئيسي؟ |  |
| **☐** | **☐** | **☐** | هل الاعتبارات الأخلاقية الموافق عليها الباحث تتطابق مع وصف الدراسة |  |
| **☐** | **☐** | **☐** | هل الهدف العلمي من البحث يبرر استخدام الحيوانات في البحث؟ |  |
| **☐** | **☐** | **☐** | هل تم تبرير أنواع الحيوانات وأعدادها؟ |  |
| **☐** | **☐** | **☐** | هل تم تبرير الجدارة العلمية لاستخدام الحيوانات؟ |  |
| **☐** | **☐** | **☐** | هل طرق التخلص من الحيوانات وفقا للمعايير الصحية والبيئية المنصوص عليها؟ |  |

**اسم المراجع/المراجعبن	رئيس اللجنة**

**التوقيع**

**التاريخ**
