# Supplementary material for: Malocclusion and orthodontic treatment needs among individuals with down syndrome in Egypt: a multicentre cross-sectional study
Source: BMC Oral Health. 2026 Oct 19;26:2060. doi: 10.1186/s12903-026-09995-9 (PMC13679113; doi:10.1186/s12903-026-09995-9)
Supplement: Supplementary file 5 [file 12903_2026_9995_MOESM5_ESM.docx]

Human Studies Application Form (PUA-REC-003)

| **Study title: Assessment of Malocclusion Patterns and Orthodontic Treatment Needs in Children with down Syndrome** | | |
| --- | --- | --- |
|  | | |
| **Source of Participants:** | 117 Patients with Down Syndrome ( age range :13-18 years) | |
| **Inclusion criteria:** | Asses the prevalence of various types of malocclusion in children with down Syndrome, Evaluate the orthodontics needs using the index of orthodontic treatment ( IOTN) | |
| **Exclusion criteria:** | Patients above 40 years | |
| **Informed consent forms attached** | the study is a Cross Sectional Observational study | |
| **Potential benefits, if any, to the participants:** | not applicable | |
| **Privacy and confidentiality of subjects are assured** | ☐ Yes ☐ No | |
| **Early withdrawal of participants (when and how to withdraw participants, data collection and follow-up for withdrawn participants):** | not applicable | |
| Study Procedures | | |
| **Study visits including procedure/tests involved at each visit (e.g., blood test, x-rays, questionnaires):** | not applicable | |
| **Follow-up evaluation:** | not applicable | |

| Please, state the following | |
| --- | --- |
| **The research is for the good of the society:** |  |
| **Facilities for the research are available:** | Clinical Examination ,Intraoral scanner,Intraoral and extraoral Photographs |
| **List the risks of the study:** | None |

☐ I declare that I will comply with the ethics of scientific research stipulated by law.

Principal Investigator , Name: Dr Hani Alswafeeri , Signature: 13-10-2025
