# Supplementary material for: Malocclusion and orthodontic treatment needs among individuals with down syndrome in Egypt: a multicentre cross-sectional study
Source: BMC Oral Health. 2026 Oct 19;26:2060. doi: 10.1186/s12903-026-09995-9 (PMC13679113; doi:10.1186/s12903-026-09995-9)
Supplement: Supplementary file 6 [file 12903_2026_9995_MOESM6_ESM.docx]

**Informed Consent Checklist (PUA-REC-006)**

**نموذج قائمة التدقيق للموافقة المستنيرة**

**عنوان البحث Assessment of**

**malocclusion patterns and orthodontic treatment needs in children with Down syndrome.**

| **نعم** | **لا** | **غير مطبق أو غير متواجد** | **ITEMS** |
| --- | --- | --- | --- |
|  |  |  | **REQUIRED ELEMENTS العناصر المطلوبة** |
| **☐** | ☐ | ☒ | المدة المتوقعة للمشاركين بالدراسة |
| **☒** | ☐ | ☐ | وصف الإجراءات الواجب اتباعها |
| **☒** | ☐ | ☐ | تحديد أي الإجراءات المبنية على التجارب (وليست ضمن الرعاية الأساسية) |
| **☒** | ☐ | ☐ | شرح للمخاطر والمضايقات المتوقع منطقياً حدوثها للمشارك بالبحث |
| **☒** | ☐ | ☐ | وصف لأية فوائد متوقعة منطقياً للمشاركين بالبحث أو غيرهم |
| **☒** | ☐ | ☐ | الإفصاح عن الإجراءات البديلة المناسبة أو الدورات العلاجية ، إن وجد ، والتي قد تكون مفيدة للمشارك |
| **☒** | ☐ | ☐ | بيان يشرح إلى أي درجة يمكن المحافظة غلى سرية وهوية سجلات المشاركين |
| **☐** | ☐ | ☒ | إذا كان البحث يخضع للوائح هيئة الغذاء والدواء فيجب إرفاق بيان يوضح احتمالية تدقيق النتائج من قبل الهيئة |
| **☐** | ☐ | ☒ | للأبحاث التي تشمل أكثر من الحد الأدنى من المخاطر، يجب إرفاق شرح ما إذا كان هناك تعويض متاح في حالة حدوث إصابة |
| **☐** | ☐ | ☒ | شرح حول ما إذا كان هناك أي علاج طبي متاح في حالة حدوث الإصابة وطبيعة هذا العلاج ، أو أين يمكن الحصول على المزيد من المعلومات بشأنه. |
| **☐** | ☐ | ☒ | تحديد وسيلة اتصال بالشخص المسؤول بالإجابة عن الأسئلة المتعلقة بالبحث وبحقوق المشاركين |
| **☐** | ☐ | ☒ | الشخص المخول بالإجابة في حالة حدوث إصابة للمشاركين بالبحث. |
| **☐** | ☐ | ☒ | بيان بأن المشاركة بالبحث تطوعية |
| **☐** | ☐ | ☒ | بيان يوضح بأن رفض المشاركة بالدراسة لا يوجب عقوبة أو حرمان المشارك من أي مزايا أو حقوق أخرى. |
| **☐** | ☐ | ☒ | بيان يوضح أنه من حق المشارك عدم الاستمرار بالدراسة وفي أي وقت دون تعرضه للعقوبة أو حرمانه من مزايا أخرى . |
| **☐** | ☐ | ☒ | بيان يوضح احتمالية أن ينطوي علاج ما أو إجراء محدد ربما ينطوي على مخاطر غير متوقعة للمشاركين بالبحث. |
| **☐** | ☐ | ☒ | مسؤليات المشارك |
| **☐** | ☒ | ☐ | هل يترتب على المشارك أية أعباء مالية جراء مشاركته بالدراسة. |
| **☐** | ☒ | ☐ | هل هناك تكلفة مسبقة يمكن التنبوء بها على المشاركين في التجربة الإكلينيكية؟ |
| **☐** | ☒ | ☐ | بيان بأن في حالة ظهور نتائج جيدة مهمة خلال البحث من شأنها أن تؤثر على رغبة المشارك في الاستمرار بالبحث فإنه سيتم إبلاغه بها. |
| **☐** | ☐ | ☒ | بيان بأنه تحت ظروف غير متوقعة يمكن للباحث أن يلغي مشاركة المشاركين بالبحث دون أي اعتبار لموافقة المشارك المسبقة. |
| **☒** | ☐ | ☐ | العدد التقريبي للمشاركين بالدراسة |
| **☒** | ☐ | ☐ | توضيح بشأن موافقة المشاركين المسبقة على استخدام عيناتهم لاحقاً لأبحاث أخرى للباحثين والمتعاونين معهم. |

**اسم المراجع/المراجعبن	رئيس اللجنة**

**التوقيع**

**التاريخ**
