# Supplementary material for: Malocclusion and orthodontic treatment needs among individuals with down syndrome in Egypt: a multicentre cross-sectional study
Source: BMC Oral Health. 2026 Oct 19;26:2060. doi: 10.1186/s12903-026-09995-9 (PMC13679113; doi:10.1186/s12903-026-09995-9)
Supplement: Supplementary file 7 [file 12903_2026_9995_MOESM7_ESM.docx]

| Study number: | |
| --- | --- |
| Researcher information: | |
| **Investigators’ information:** |  |
| **Principal Investigator:** | Hani AlSwafeeri |
| Title /Position | Lecturer of Orthodontics |
| Affiliation: | Pharos University |
| Department: | orthodontics |
| Mailing Address: | Pharos University – Canal ElMahmoudia - Smouha - Alexandria |
| Email: | Hani.alswafiri@pua.edu.eg |
| Cell- Phone: | 01001830264 |

Researcher Application form (PUA-REC-001)

| Members of the research team involved in the design, conduct, or reporting of the research | | | |
| --- | --- | --- | --- |
| Name | Title / Affiliation | Contact Information Email/Telephone | Role in the research |
| Hani AlSwafeeri | Lecturer of Orthodontics | Hani.alswafiri@pua.edu.eg | Supervising clinical steps and cast measurements , revision of manuscript |
| Nancy Bedweni | Lecturer community of Dentistry | Nancy bedwani @pua.edu.eg | Supervising clinical steps and writing mauscript |
| Mariam Saaied Badran | Senior student | 202102082@pua.edu.eg | Performing IOTN and Clinical analysis |
| Nada Zakaria Shehata | Senior student | 202100160@pua.edu.eg | Performing IOTN and Clinical analysis |
| -Hala Ahmed  -Menna Allah mohamed Hassan | -Senior student  -Senior Student | [202100006@pua.edu.eg](mailto:202100006@pua.edu.eg)  202101371@pua.edu.eg | Performing IOTN and Clinical Analysis |

| Study information: | |
| --- | --- |
| **Study Title:** | **Assessment of malocclusion Patterns and orthodontic treatment needs in children with Down syndrome** |
| **Category of the study:** | ☒Research ☐ Master student ☐ PhD student ☐ Project ☐Training |
| **Duration of approval requested:** | ☐ 1 Month |
| **Type of submission:** | ☒ New ☐ Re-submission ☐others  Pre-submission number:  Others: |
| **Study Area:** | : analysis of records |
| Funding Sources (if any) | |
| **Funding source:**  Self Funded | |
| **Investigator financial conflicts of interest, if any:**  None | |

☒I declare that I will comply with the ethics of scientific research stipulated by law.

Principal Investigator	Name: Hani Alswafeeri	Signature. Date: 13/10/2025
